# Case report: Spinal cord stimulation in the treatment of pediatric erythromelalgia

**DOI:** 10.3389/fneur.2023.1143241

**Published:** 2023-05-18

**Authors:** Lei Zuo, Ai Su, Ying Shi, Nan Li, Shiyu Chen, XiaoQiu Yang

**Affiliations:** Department of Pain, The First Affiliated Hospital of Chongqing Medical University, Chongqing, China

**Keywords:** case report, spinal cord stimulation, pediatric erythromelalgia, multidisciplinary approach, neuromodulation

## Abstract

**Introduction:**

In children, erythromelalgia is a rare chronic pain syndrome characterized by erythema, severe burning pain, and itching of affected feet. Unfortunately, there is no definitive therapy available currently.

**Case report:**

Here, we report a case of primary erythromelalgia and the treatment response in a 10-year-old boy, whose genetic findings for mutations in the SCN9A gene were positive and skin biopsy results were diagnosed as small fiber neuropathy, while he has suffered from excruciating burning pain, itching, erythema, and recurrent infections over the past 3 years. He did not respond well to conventional treatment, and the only way to receive minimal relief was to immerse his feet in ice water. After a successful trial of spinal cord stimulation (SCS), the implantable pulse generator (IPG) was successfully implanted without complications, and it proved partial response to therapy.

**Conclusion:**

There is no specific, efficient treatment for pediatric erythromelalgia currently, but this case demonstrates neuromodulation serves as part of the multimodal regimen to treat pediatric erythromelalgia.

## Introduction

Erythromelalgia was first named in 1878 by Mitchell (1), and later categorized as a primary (which may be sporadic or familial) or secondary form with different etiology by Smith and Allen in 1938 (2). Symptoms of primary erythromelalgia (PE) typically start in childhood and it may develop and last with age. It is characterized by intense burning pain that is accompanied by erythema, itchiness, and swelling. Standing, exercise or localized heat exposure can make it worse, while cooling down or immersion in ice water can make it better (3). Although the exact etiology of PE remains unknown, genetic research has identified it is primarily brought on by gain-of-function mutations in the SCN9A gene coding for the subtype Nav1.7 voltage-gated sodium channel (4, 5). It is mainly manifested in expressed in the sympathetic and nociceptive small-diameter sensory neurons of the dorsal root ganglion that contribute to the pain. A series of research on PE have elucidated that the site of mutation can influence both the abnormality of Nav1.7 channel function, age of onset and severity of symptoms (6–9): There are polymorphisms of the gene mutation that increase the severity of more complex pain phenotypes. Changes in sympathetic neuron function result in microvascular symptoms, and nociceptive neuron function led to severe burning pain, characterizing erythromelalgia (10). Secondary erythromelalgia is not associated with any specific genetic mutations but is associated with various underlying conditions including hematological diseases, myeloproliferative disorders, infections, neoplastic diseases, metabolic diseases and medications (11). Unlike the primary type, secondary type pathophysiology is poorly understood and thought to be due to neuropathological and microvascular functional changes caused by the underlying condition. Thus, the reasons for the poor treatment status are multiple, mainly due to the complex etiology and unknown mechanism of action of erythromelalgia.

Additionally, there are no vivid clinical guidelines or consensus on the management of pain in PE, as well as the unclear mechanism, which makes the clinical treatment of PE fraught with difficulties, and highly variable responses to therapy. Even though multiple approaches have been explored with varying degrees of success, previous studies have revealed that most cases are refractory to traditional pharmacologic therapy (12), including gabapentinoids, tricyclic antidepressants, serotonin-norepinephrine reuptake inhibitors, calcium channel blockers, opioids, and topical drugs. Similarly, interventional procedures such as sympathetic blocks and epidural infusion may only be partially effective. As a result, the treatment regimen of this channelopathy with resultant neuropathic pain should follow a multidisciplinary approach.

As we all know, SCS has been widely adopted for various intractable pain syndromes, such as failed back surgery syndrome, herpetic neuralgia, complex regional pain syndrome, and refractory radiculopathy (13–15). The first instance of SCS being used to effectively manage pain in a patient with erythromelalgia was reported in 1993 (16). However, a few cases have been published to assess the effectiveness of neuromodulation to treat this disorder over the past 30 years. Recently, studies have demonstrated that SCS may have a critical role in successful treatment (17–20). It's possible to undervalue the therapeutic benefits of SCS in PE. Here, we present the case of a 10-year-old PE patient who responded favorably to SCS treatment.

## Case report

**Figure 1:**
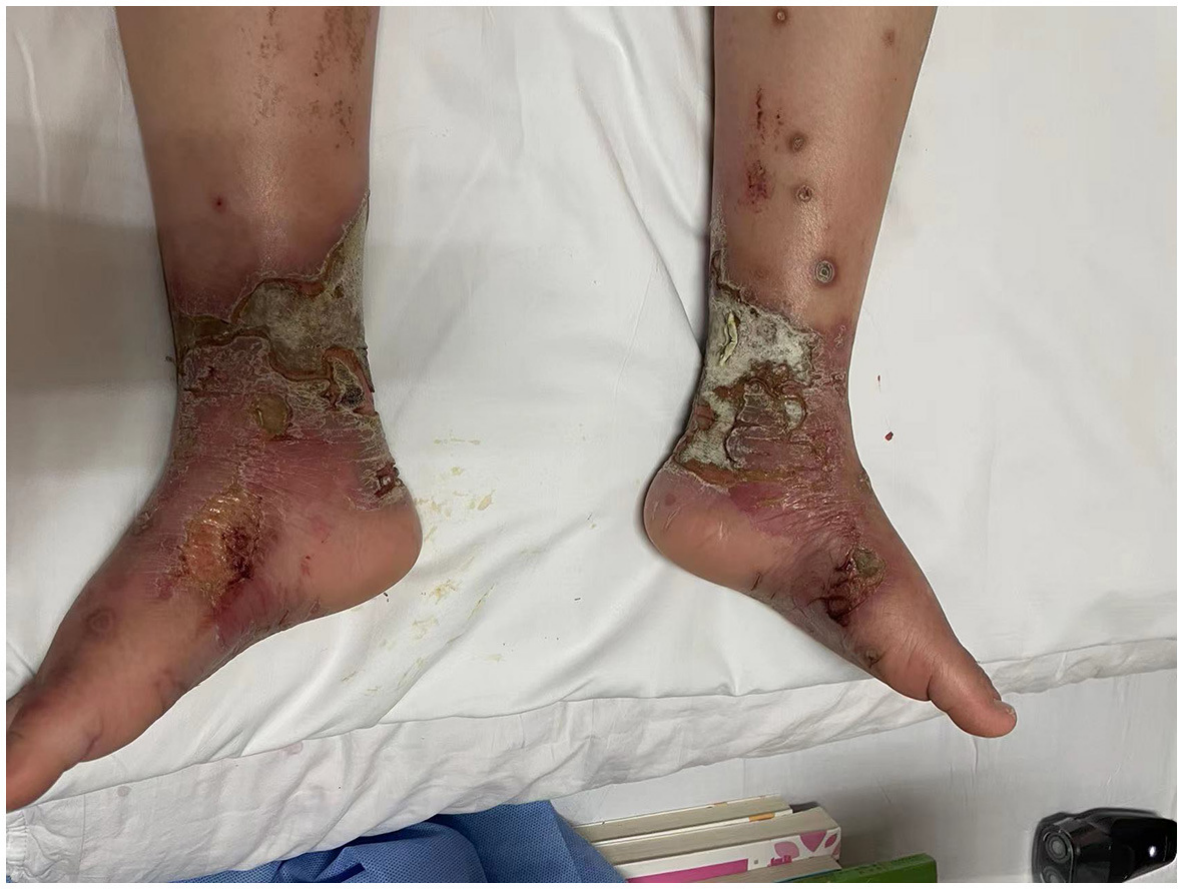
Erythromelalgia complicated by immersion foot syndrome and infection: bullae, erosions, painful ulcers, and infection on the patient's feet and ankles.

This case is reported with the written consent of the patient and his guardian. A 10-year-old, 45-kg Asia boy was diagnosed with small fiber neuropathy and erythromelalgia at the age of 7. The patient recalled that he experienced a sudden onset of pain in both feet, while the pain was aggravated by heat, exercise, and walking. The whole blood samples from the patient and his parents have performed the gene detection at that time, the sequencing results of the patient showed the presence of heterozygous variation of SCN9A gene C.706G>T (p.G236W), while no related variation was detected in his parents, suggesting this variation may be *de novo* mutation. Further skin biopsy was utilized to confirm the diagnosis of small fiber neuropathy. He had undergone treatment from a dermatologist and a neurologist for pain that had initially been restricted to his feet over the previous 3 years. Although he had been taking medications regularly for a year, such as Mexiletine, Oxcarbazepine, Gabapentin, mecobalamin and Carbamazepine, they had a significant negative impact on his liver function and caused an illness to develop and worsen for the previous two years. He described the pain as a “burning pain,” “hot sensation,” and “itching sensation,” while the pain located in his feet was measured using a visual analog scale (VAS) with a score of 6. It is difficult for patients to wear socks or shoes and to attend school or socialize with friends. He needs to rub his feet repeatedly and need to submerge his feet in ice water multiple times to get relief. Consequently, these behaviors impair skin integrity and exacerbate microcirculatory abnormalities, leading to bullae, erosions, painful ulcers, and recurrent infections (Figure 1).

**Figure 2:**
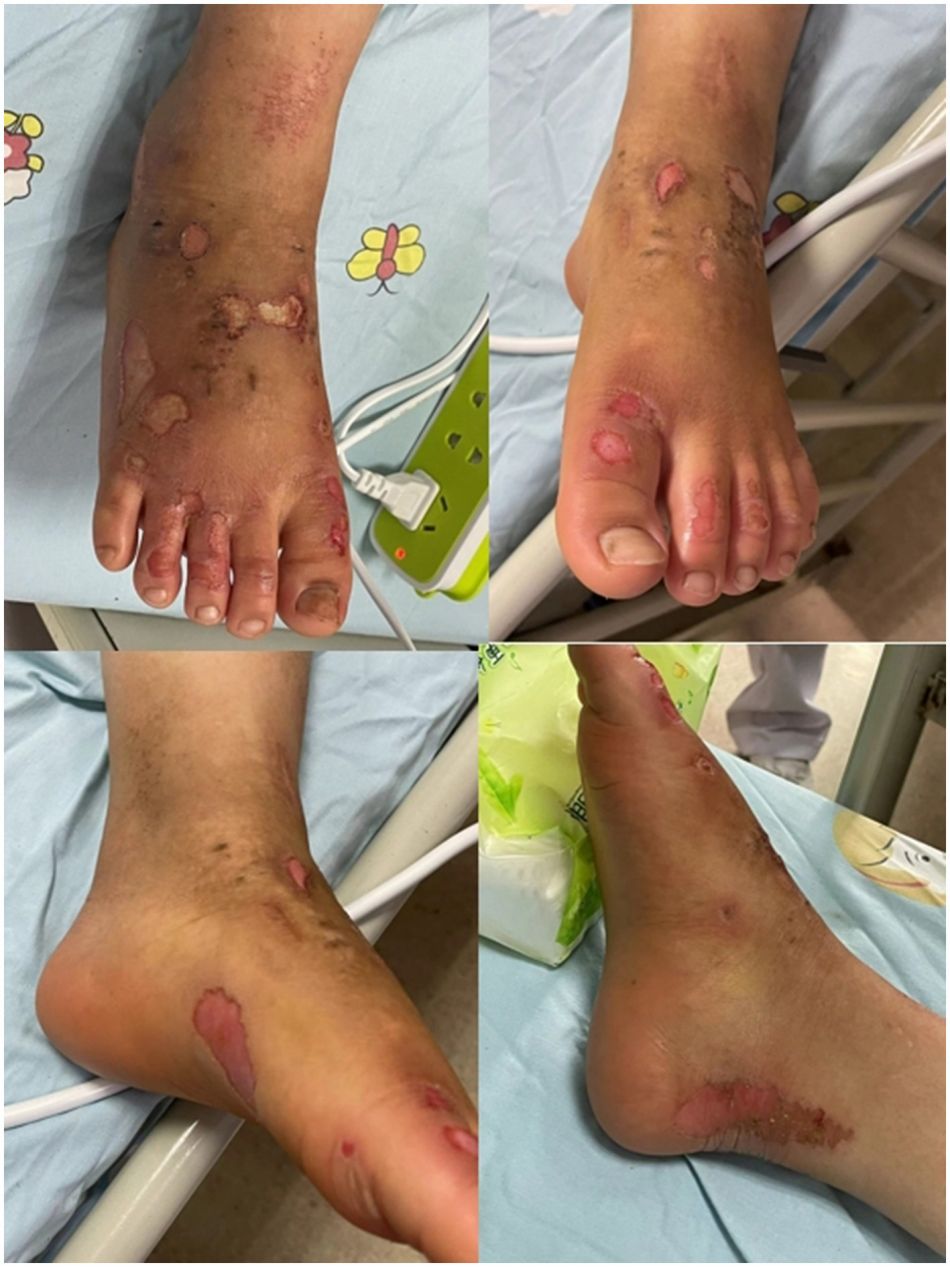
The appearance of lower extremities at the first hospitalization.

A pain consultation was obtained during his hospitalization in the dermatology department of the children's hospital for lower extremity infections and severe burning pain (Figure 2). The patient had attempted several analgesic treatments, including sufentanil and another intravenous analgesia (tramadol, morphine) infusion, carbamazepine (400 mg twice per day), gabapentin (200 mg three times per day), mexiletine (100 mg twice per day), amitriptyline (25 mg daily), neurotrophin (200 mg twice per day), mecobalamin (0.5 mg twice per day), aspirin (50 mg once a day), lidocaine cream, befuxin and amikacin, which proved no significant improvement. Therefore, he was transferred to our pain management department (Figure 2). After a discussion with her parents, an epidural catheter was placed at the L2–L3 level under local anesthesia. Five milliliters of 0.3% lidocaine were administered via bolus injection, followed by an infusion of 0.15% Lidocaine at 2 mL/h for 5 days. On the VAS score, the patient scored 0 for pain. He no longer needed to immerse his feet in cold water. The mexiletine was gradually withdrawn, the carbamazepine was adjusted from 400 mg bid to 200 mg tid, and gabapentin 200 mg tid was reduced to 100 mg tid.

**Figure 3:**
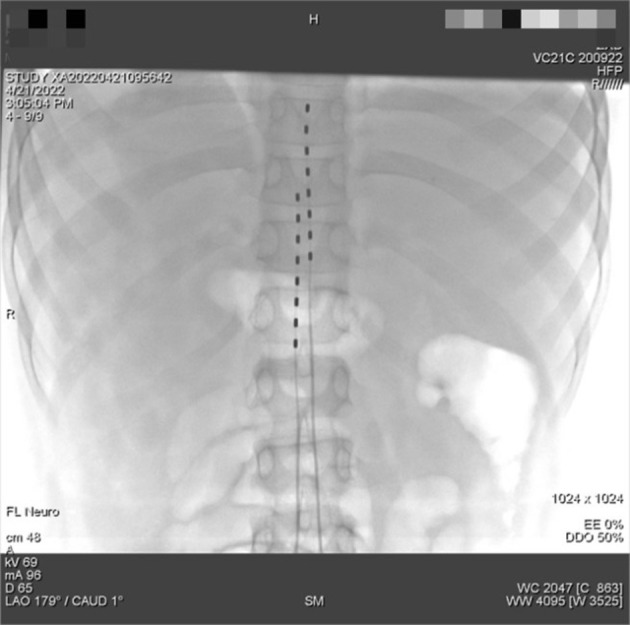
Inoperative digital subtraction angiography imaging for the placement of the dual-lead spinal cord stimulator placement. The tip of the 2 spinal cord electrical stimulation electrodes were placed in the epidural space near the upper edge of the T11 and T9 vertebral body.

**Figure 4:**
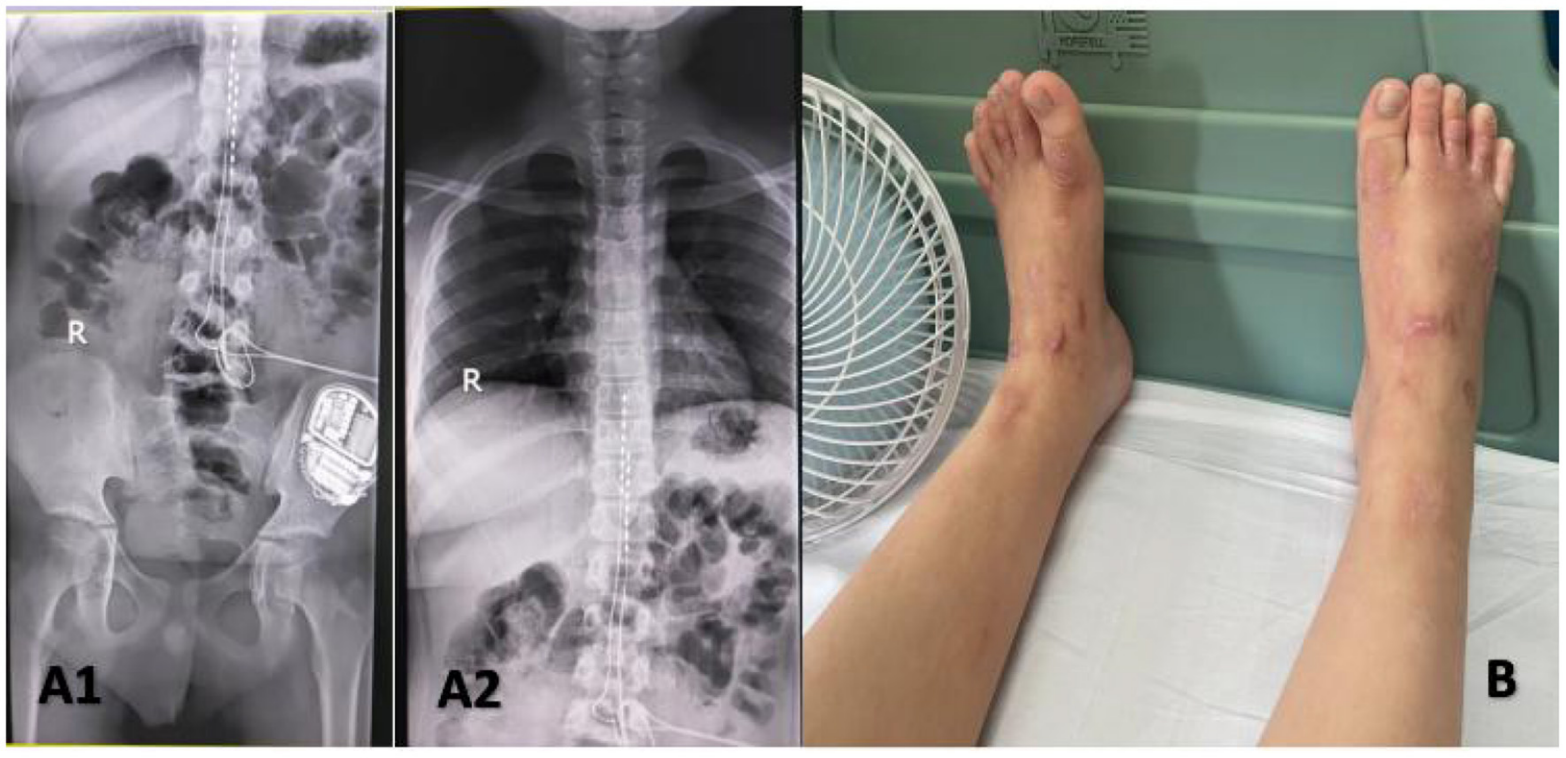
Implanted a permanent Medtronic intellis neurostimulator **(A1, A2)**. Appearance of lower extremities after dorsal column stimulation **(B)**.

**Figure 5:**
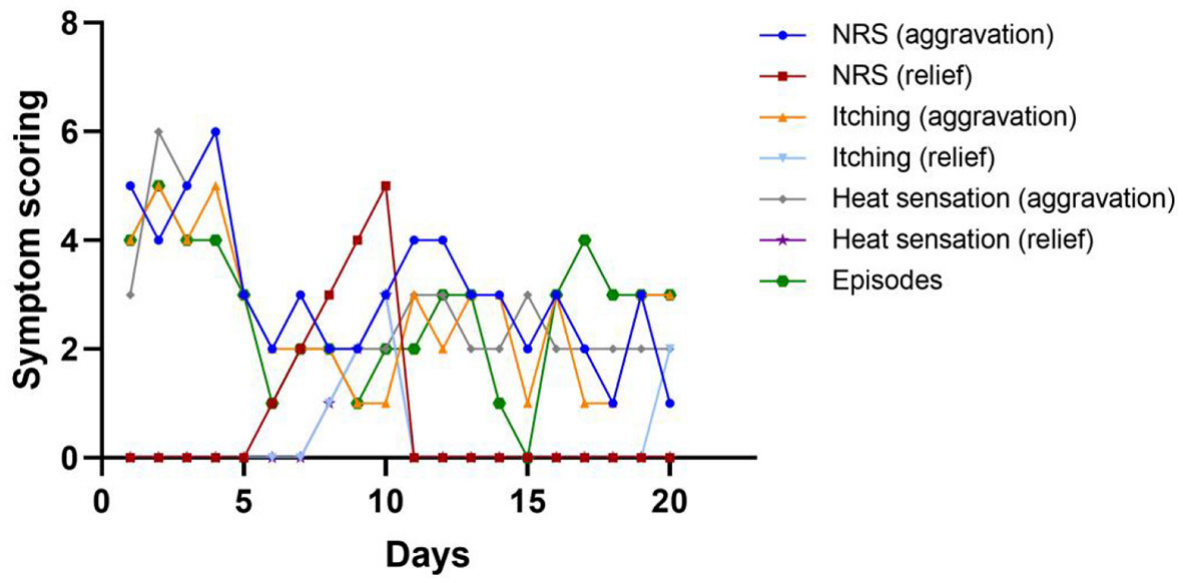
Line chart of symptom scoring and episodes of this patient during hospitalization. Among these days, he received stage I implant operation at day 1, and stage II implant operation at day 14.

However, the duration of the curative effect only lasted for about a month, and the symptoms began to recur and grew worse. The alternation of strong itching and insupportable pain causes emotional upset, as well as sleep disturbances. During his second hospitalization, combined with multi-disciplinary consultations, we tried to help the patient to establish an appropriate cooling strategy and adjusted pharmacologic treatments. Based on the consultation of the pharmacy, amitriptyline was changed to doxepin hydrochloride (25 mg) and cyproheptadine cream to relieve the refractory pruritus; fentanyl transdermal patches were given to suppress the pain, besides, multiple nerve-blocking treatments including tibiofibular nerve block and lumbar epidural nerve block were performed, but his symptom did not obtain a satisfactory improvement. Lidocaine (50 mg, i.v., qd) was gradually increased to 90 mg for pain control. Concerning the consultation of neurology, the nerve growth factor, gabapentin and mexiletine were applied. Besides, the alprazolam hydrate 1.344 g was utilized to improve sleep quality, with psychological counseling and health education based on the consultation of psychiatry. According to the consultation of dermatology, glycyrrhizin 25 mg was used to ameliorate the pruritus, and polymyxin B was used as the external application for wound treatment. We also consulted the burns department, and the wound crusting removal and external application of fibroblast growth factors were given. However, the therapeutic approaches did not reach a satisfactory outcome. Given his refractory case, SCS was offered to him. The SCS therapy was approved and recorded by the ethics committee of hospital. The patient and his parents agreed to a trial. SCS electrodes were placed in the epidural space at the level of the 11 (right) and 9 (left) vertebral body under the guidance of digital subtraction angiography (Figure 3), which provided complete coverage of the patient's feet and calves during the operation and post-operative days. The trial lasted 13 days, the patient's VAS score decreased from 2 to 3 out of 10 during this period, the number of paroxysms was gradually reduced from the initially more than 10 times to 1–3 times, and the duration of pain has been inhibited from 30 to 10–20 min. Besides, the itching sensation has largely been alleviated, accompanied by the remarkable improvement of emotional upset and sleep disturbances. His functional status was ameliorated as well. As the patient's symptoms and function continued to improve significantly, we successfully implanted a permanent Medtronic intellis neurostimulator (Medtronic, 97716) with no complications. After 6 days of neurostimulator implantation, the appearance of the skin gradually recovered (Figure 4). In the process of setting the electrical stimulation parameters, we found it interesting that different frequencies affected the patient differently (Table 1). We subsequently tried to set various stimulation parameters with the range from 60 to 1,200 Hz. The results showed that the itching, pain, and burning sensation were alleviated to varying degrees, however, it cannot completely improve all his symptoms (Figure 5). The patient was discharged with only a small dose of medication.

**Table 1:** Effect of the different SCS stimulation frequencies on symptoms.

**Unilateral electrical stimulation (frequency, Hz)**	**Effects on symptoms**
1,200	Pain was released, with itching and heat sensation
600	Obvious heat sensation, but no obvious itching and pain
500	Peripheral vascular dilation, with obvious heat sensation
300	Weakened heat sensation with significant itching, but no obvious pain
50	Obvious itching, but no obvious heat sensation

At the 2-month follow-up, because of the continuous high temperature, the patient developed a skin infection and ulcers after soaking his feet in ice water, resulting in recurrent pain. Through skin treatment, rehabilitative education, drug adjustment, and continuous SCS therapy, the frequency of foot pain in the latest follow-up was relieved compared with the previous condition. The patient has no significant episodes of pain during the day and has one or two episodes of increased pain at night. After soaking both feet in cold water for about 30 min, the pain was relieved. SCS did not reduce the itching although it relieved his pain. We will continue to follow the progress.

## Discussion and conclusion

PE is a challenging, difficult-to-control disease that is largely based on a small number of cases and unclear mechanisms, as is the case in this report. Several factors limit the systematic approach to the management of the disease. First, erythermalgia is a rare disorder, estimated to affect 0.36–2 per 100,000 people per year in the USA and Europe (21, 22), making it a difficult Research Topic. Second, the underlying pathophysiology remains unclear, thereby limiting the intentional treatment of pain. Third, there is no guidance or expert consensus on this disease for reference. The long-term prognosis of patients whose treatment was ultimately effective in the case reports is unknown. Fourth, different patients may have various curative effects on the same treatment, which may be related to the sites of SCN9A gene mutation. For instance, carbamazepine and mexiletine both show efficacy in the context of specific mutations (22). Therefore, the response to treatment is dependent on the specific type of Nav1.7 mutation. There is a varying response to pharmacotherapy in this clinical population, resulting in a stepwise trial-and-error approach (23). Finally, it's logical to use medications with known broad activity against voltage-gated sodium channels, such as anticonvulsants and local anesthetics. Due to adverse effects on the cardiac and central nervous system, the therapeutic window is narrow. The development of selective blockers of Nav1.7 sodium channels is in the clinical trial stage (24). Unfortunately, many patients continue to see little benefit from the treatments that are currently available, despite promising studies that may shed light on potential future interventions.

SCS is often used for refractory chronic pain diseases in clinical practice (25). Although few cases have been reported on the treatment of erythermalgia by SCS, the treatment results in these reports are consistent in that SCS can cure or partially improve those symptoms, especially painful sensations. Although it has been not clear why there are such differences in treatment at present, we found that different frequencies of stimulation to the boy will produce various clinical effects. Without the patient's knowledge, we set a constant pulse width and voltage (pulse width 180–200 us, voltage 2.0–4.0 V) with different frequency parameters. Set to 1,200 Hz: Only unilateral electrical stimulation was used. After walking, the pain on the open electrical stimulation side was significantly improved; Set to 600 Hz: no obvious itching on both feet, but obvious heat sensation on both feet; Set to 500 Hz: more consistent with peripheral vascular dilation; Set to the 300 Hz: weakened heat sensation with significant itching; Set to 50 Hz: obvious itching (Table 1). However, we failed to find a suitable stimulus parameter to improve all his symptoms. When electrical stimulation is used to treat other diseases, it also has different therapeutic effects at different frequencies. For example, Li et al. (13) displayed that 60–80 Hz may be useful for axial symptoms (e.g., gait, freezing, and speech) when electrical stimulation is used to treat Parkinson's disease, while high frequency (>100 Hz) can significantly improve the cardinal motor symptoms (i.e., tremor, rigidity, and bradykinesia). It is well-accepted that sensory fibers have different diameters depending on the type of sensory information they encode (26, 27). For instance, pain fibers transmitting burning sensation (c-fiber type) exhibit the smallest diameter, while A-delta fibers transmitting itching sensations have a larger diameter compared to the C-fibers. We speculated that there might be a relationship between the diameter of the nerve fibers and the SCS frequency, generally, larger-diameter sensory fibers transmit information more quickly than smaller-diameter fibers (28). This phenomenon is known as frequency-dependent recruitment. Additionally, the itch sensation is primarily transmitted by unmyelinated type C and thinly myelinated type A-delta nerve fibers in the skin. The cell bodies of these nerves reside in dorsal root ganglia with axons innervating the skin and dendrites synapsing in the dorsal horn of the spinal cord. There is a complex interplay between these nerve fibers and receptors on these sensory nerve endings, which activate Nav1.7 and Navl.8 sodium channels (29), propagating the action potential of the itch signal. It is well-confirmed that PE is related to the abnormality of Nav1.7 channel function, which may be caused by the site of mutation, hence, the typical clinical manifestation of itching in this patient could be explained by this hypothesis, and neuro-modulation can improve this symptom. Collectively, neuromodulation plays a potentially key role in the treatment of PE, while future exploration of additional stimulation modalities may offer new hope for the treatment of erythromelalgia.

Furthermore, it is crucial to engage the patient and family in physical and psychological therapies to optimize function, as well as pain reduction. Substantial education was necessary to produce a behavior change. There is a central reason that impaired skin integrity and microcirculatory abnormalities from overexposure to water and cold can result in ulceration, maceration, and infections (30). In this case, the patient also suffered repeated foot infections since he did not establish proper cooling strategies. Therefore, patients need to be counseled on the appropriate use of cooling strategies, balancing tolerance of pain, and functional activities.

Although SCS did not completely improve all symptoms of patients as we expected in this case, it is superior to conventional medical management for several similarly difficult-to-treat pain conditions. Interestingly, this study also found that different frequencies may have improved different symptoms in this boy, and future exploration of additional stimulation modalities may offer new hope for the treatment of erythromelalgia. This case spurs interest in future research in neuromodulation as part of the multimodal regimen to treat pediatric erythromelalgia.

## Data availability statement

The original contributions presented in the study are included in the article/Supplementary material, further inquiries can be directed to the corresponding author.

## Ethics statement

The studies involving human participants were reviewed and approved by the Ethics Committee of the First Affiliated Hospital of Chongqing Medical University. Written informed consent to participate in this study was provided by the participants' legal guardian/next of kin.

## Author contributions

LZ: drafting and revision of the manuscript, management and follow-up of the patient, and application for ethical review. XY: critical revision of the manuscript for intellectual content and application for ethical review. AS: management and follow-up of the patient. YS and NL: management the patient. SC: follow-up of the patient and interpretation of the data. All authors contributed to the article and approved the submitted version.
