# Supplementary figures and images for: Case report: Spinal cord stimulation in the treatment of pediatric erythromelalgia
Source: Front Neurol. 2023 May 18;14:1143241. doi: 10.3389/fneur.2023.1143241 (PMC10233004; doi:10.3389/fneur.2023.1143241)

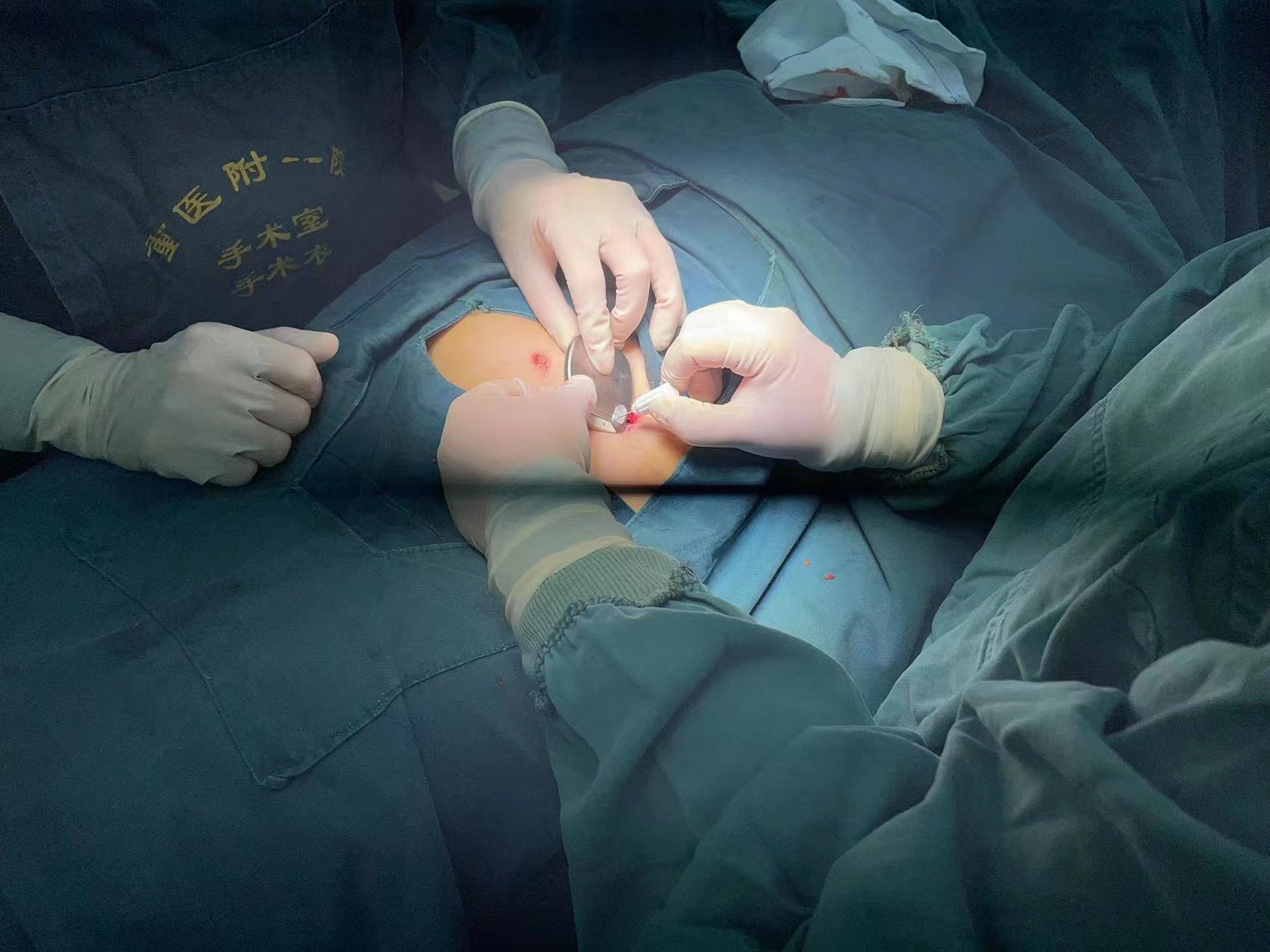

Supplement: Supplementary file 1 [file Image_1.JPEG]

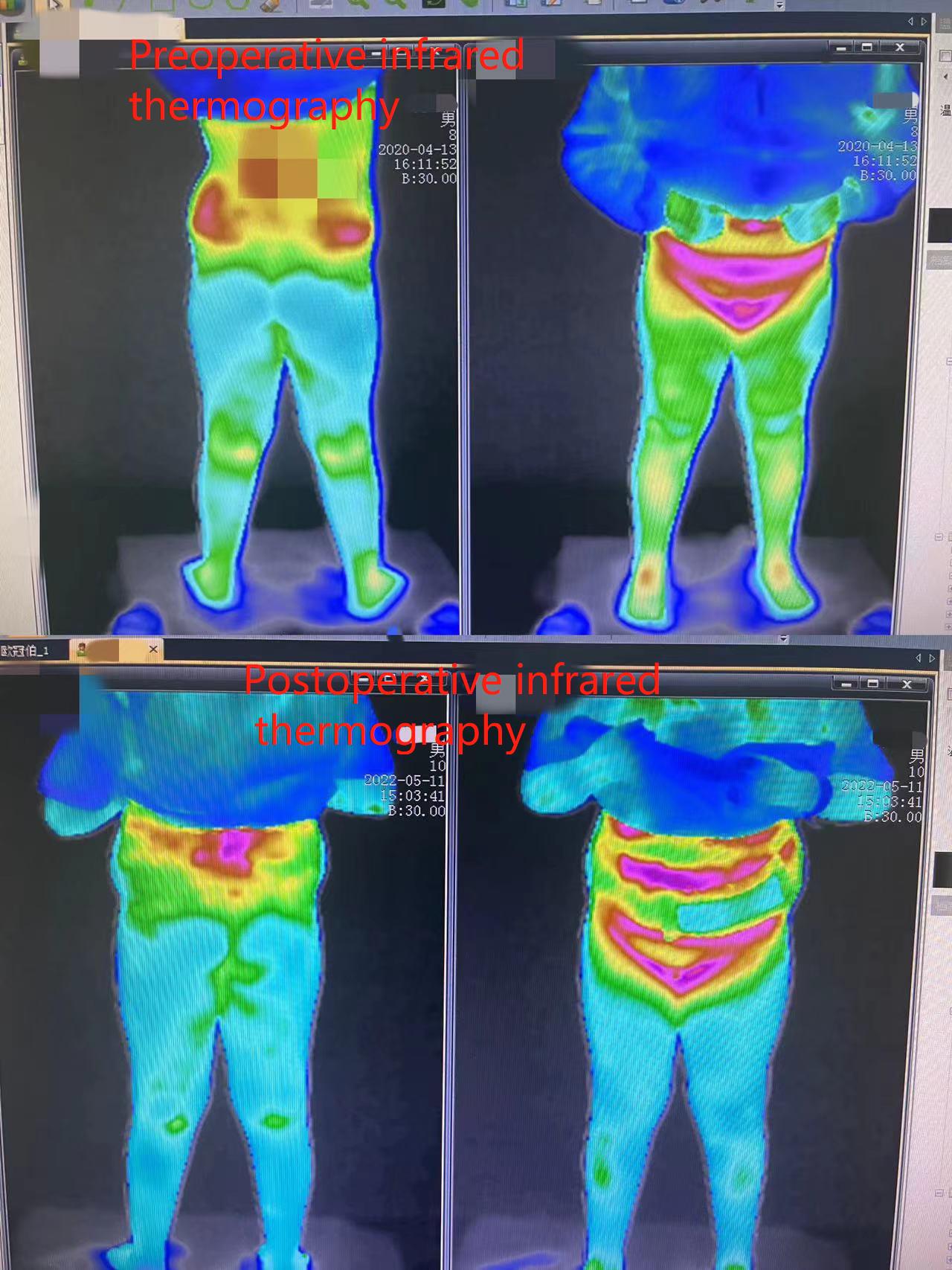

Supplement: Supplementary file 2 [file Image_2.JPEG]

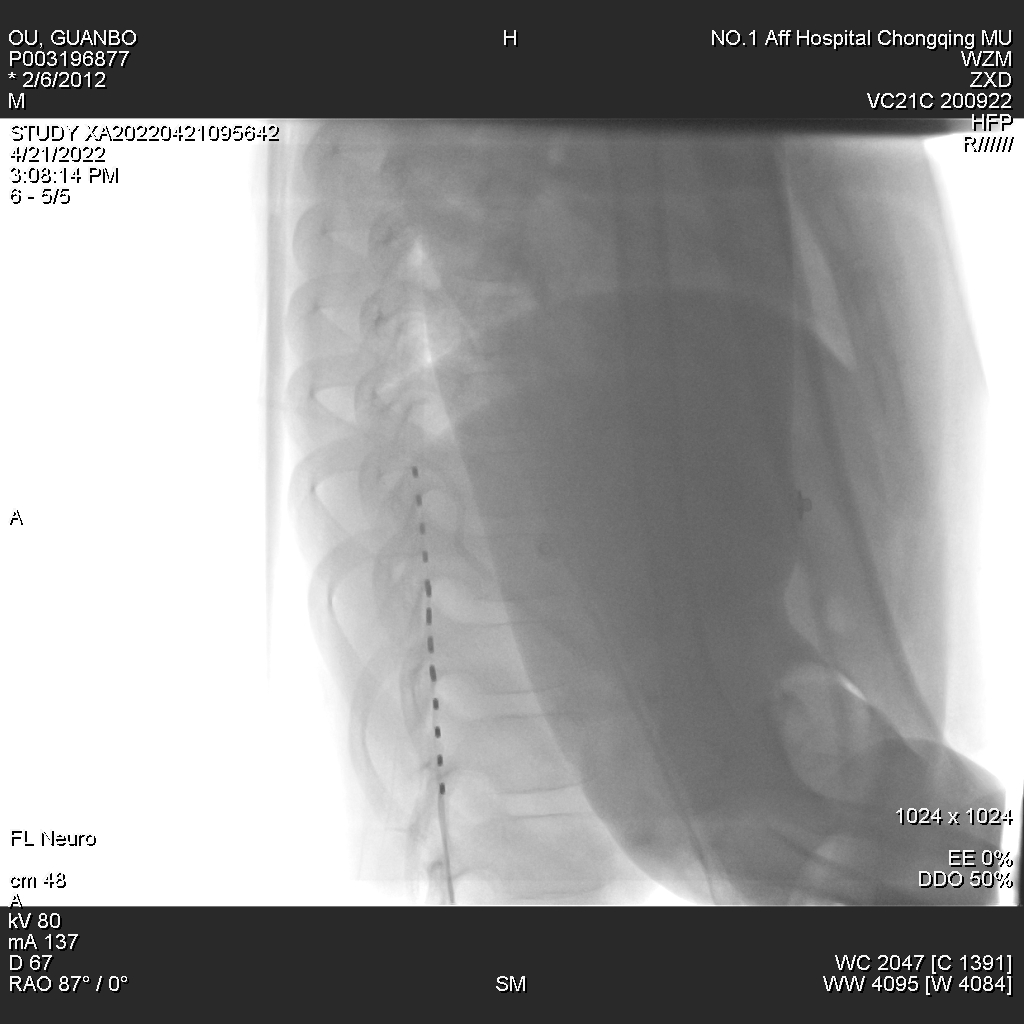

Supplement: Supplementary file 3 [file Image_3.JPEG]

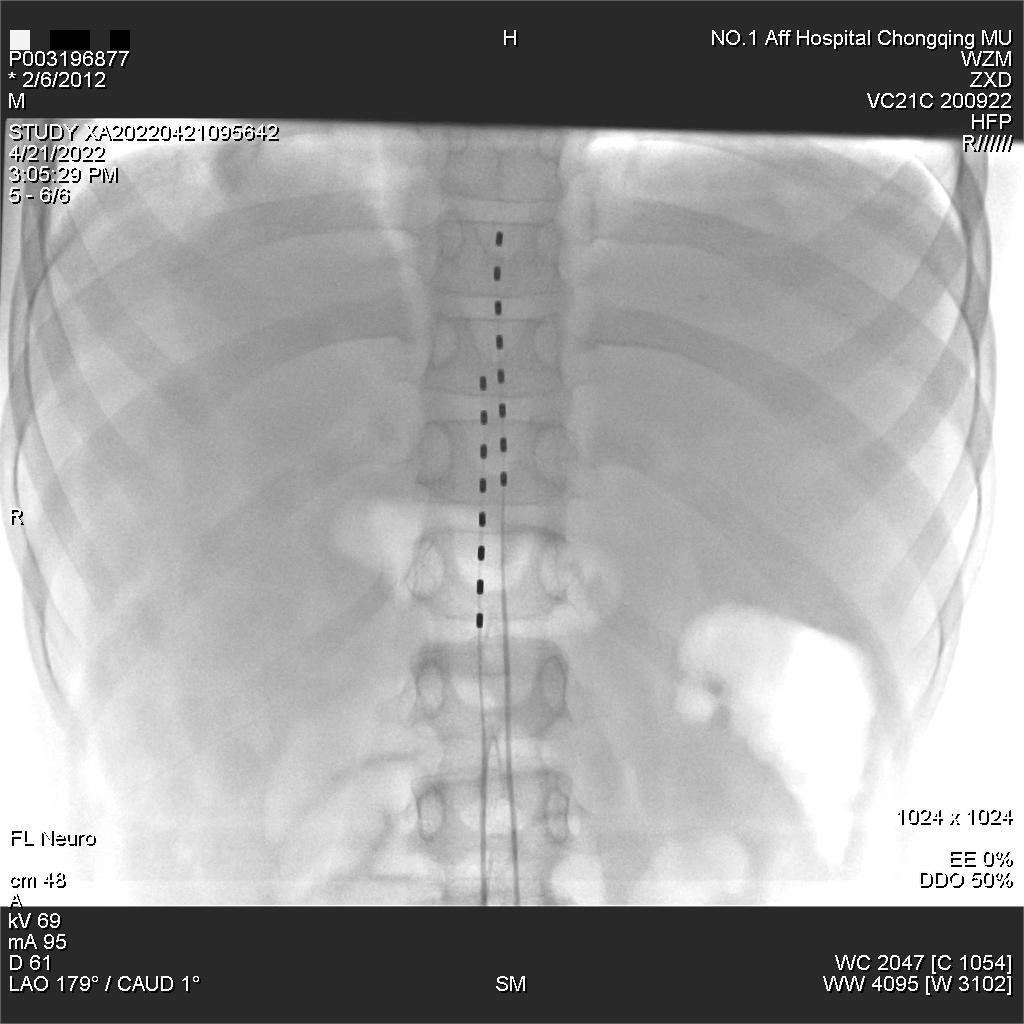

Supplement: Supplementary file 4 [file Image_4.JPEG]
